# Genetic diversity and distribution dynamics of multidrug-resistant *Mycobacterium tuberculosis* isolates in Nepal

**DOI:** 10.1038/s41598-018-34306-w

**Published:** 2018-11-09

**Authors:** Bhagwan Maharjan, Chie Nakajima, Norikazu Isoda, Jeewan Thapa, Ajay Poudel, Yogendra Shah, Tomoyuki Yamaguchi, Bhabana Shrestha, Harald Hoffmann, Korkut Avsar, Ashish Shrestha, Stephen V. Gordon, Yasuhiko Suzuki

**Affiliations:** 1 German Nepal TB Project (GENETUP/NATA), Kathmandu, Nepal; 2 Division of Bioresources, Hokkaido University, Research Center for Zoonosis Control, Hokkaido, Japan; 3 Global Institution for Collaborative Research and Education (GI-CoRE), Hokkaido University, Hokkaido, Japan; 4 Unit of Risk Analysis and Management, Research Center for Zoonosis Control, Hokkaido, Japan; 5 Department of Microbiology, Chitwan Medical College Teaching Hospital, Chitwan, Nepal; 6 WHO-Supranational Reference Laboratory of Tuberculosis, Munich Gauting, Germany; 7 KuratoriumTuberkulose in der Welt e.V, München-Gauting, Germany; 8 Asklepios Klinik, Gauting, Germany; 9 National Tuberculosis Center, Thimi, Bhaktapur, Nepal; 10 World Health Organization, Kathmandu, Nepal; 11 UCD School of Veterinary Medicine, University College Dublin, Dublin, D04 W6F6 Ireland

## Abstract

Multidrug-resistant tuberculosis (MDR-TB) is an emerging public health problem in Nepal. Despite the implementation of a successful TB control program in Nepal, notifications of MDR-TB are increasing, yet the reasons are unknown. The objective of this study was to understand the genetic diversity and epidemiological characteristics of MDR-*Mycobacterium tuberculosis* (MTB) isolates in Nepal. We isolated and genotyped 498 MDR-MTB isolates collected from April 2009 to March 2013 and analyzed the patients’ background information. Our results showed that the lineage 2 (Beijing family) was the most predominant lineage (n = 241; 48.4%), followed by lineage 3 (n = 153, 30.7%). Lineage 4 was the third most prevalent (n = 73, 14.5%) followed by lineage 1 (n = 32, 6.4%). The lineages were significantly associated with geographic region, ethnic group, age and sex of patients. The Beijing genotype was found to have an important role in transmitting MDR-TB in Nepal and was significantly associated with the eastern region, mongoloid ethnic group and younger age group. We conclude that early diagnosis and treatment including molecular-epidemiological surveillance of MDR-TB cases will help to control transmission of MDR-TB in Nepal.

## Introduction

Multidrug-resistant tuberculosis (MDR-TB) is caused by *Mycobacterium tuberculosis*(MTB) resistant to both isoniazid (INH) and rifampin (RIF), the two most effective first-line anti-TB drugs^1^. In 2016, the World Health Organization (WHO) recognized drug-resistant TB as a continuing threat with an estimated 600,000 new TB cases with RIF resistance, of which 490,000 cases were MDR-TB. Almost half of the above MDR-TB cases were in India, China and the Russian Federation^1^.

Nepal, situated in between India and China, the two countries with the greatest TB burden, also experiences many TB cases every year. In 2016, Nepal reported an estimated TB incidence and mortality of 154 and 22, respectively, per 100,000 inhabitants^1^. The Nepal government’s National TB Program (NTP) has recognized MDR-TB to be a great challenge to the national TB control program and a major public health concern in Nepal^2^. The Nepalese 2011/12 MDR-TB survey showed MDR-TB to be 2.2% in new and 15.4% in retreatment TB cases^2^. Despite Nepal having an effective TB control program under the supervision of the WHO, notification of MDR-TB was found to be increasing year on year, with the number of new cases of MDR-TB increasing consecutively for five fiscal years from 2011 to 2015^2^. In the Nepal National TB Program (NTP) annual report 2014/15, out of an estimated 1,000 MDR-TB cases, only 379 cases were diagnosed and treated^2^, suggesting substantial limitations in the management of MDR-TB, especially outside the capital city of Kathmandu valley. Furthermore, a high rate of primary quinolone resistance among MDR-TB cases is also increasing^2,3^. The reasons for the emergence of MDR-TB in Nepal are not clearly understood.

Under the supervision of NTP, cases of DR-TB are managed by DR-TB treatment centers distributed throughout the country. From Kathmandu, the NTP’s national TB center (NTC) and the German Nepal TB project (GENETUP) provides culture and drug susceptibility testing (DST). Treatment of DR-TB is performed under the NTP guidelines^2^ which entails administration of standardized treatment regimens. All MDR-TB patients receive 20 months of the standard regimen with 8 months of intensive phase involving kanamycin, levofloxacin, pyrazinamide, cycloserine and ethionamide, with a 12 month continuation phase using the same drugs except kanamycin.

Genotyping of MTB strains is important to understand their molecular epidemiology and transmission dynamics. Among commonly used genotyping methods, spoligotyping is a rapid and convenient method that can simultaneously detect and differentiate lineages of MTB^4^. Using spoligotyping the global population of MTB can be classified into four main lineages, lineage 1(Indo-Oceanic lineage), lineage 2 (East-Asian lineages, that includes Beijing family), lineage 3 (East African Indian that includes Central Asian Strain, CAS/Delhi family) and lineage 4 (Euro-American family)^5^. Previous studies have demonstrated a relationship between drug resistance and genotype of MTB^6–8^, while other studies failed to find an association^9^. The Beijing genotype is reported to have unusual characteristics such as hyper-virulence, higher pathogenicity, a greater ability to overcome immunity afforded by BCG vaccination and frequent association with MDR-TB^10,11^. Previous studies from Nepal^12,13^ have demonstrated high genetic diversity of MTB isolates. In the study conducted from 2009 to 2010^12^, the authors described the first phylogenetic diversity of MTB isolates in Nepal with the CAS/Delhi family being the most dominant (40.6%, 106/261), followed by Beijing family (32.2%, 84/261). However, most of the isolates were non-MDR (245/261) and were mostly from the Kathmandu valley. Importantly, in a few MDR isolates (16/261), most isolates (56.3%, 9/16) were of the Beijing family followed by the CAS family (6/16, 37.5%), suggesting the need for further studies on genetic diversity of MDR-MTB isolates in Nepal. However, there has been no comprehensive molecular epidemiological study of MDR-MTB isolates to aid in our understanding of the emergence of MDR-TB in Nepal.

To understand the emerging public health problem of MDR-TB in Nepal, we genotyped 498 MDR-MTB isolates collected from all geographic regions of the country between April 2009 and March 2013. The aim of this study was to gain insights into the genetic background of these selected MDR-MTB isolates and any association with the TB patients’ background information so as to understand the factors associated with the emergence MDR-TB in Nepal.

## Results

### Characteristics of MDR-MTB isolates

**Figure 1:**
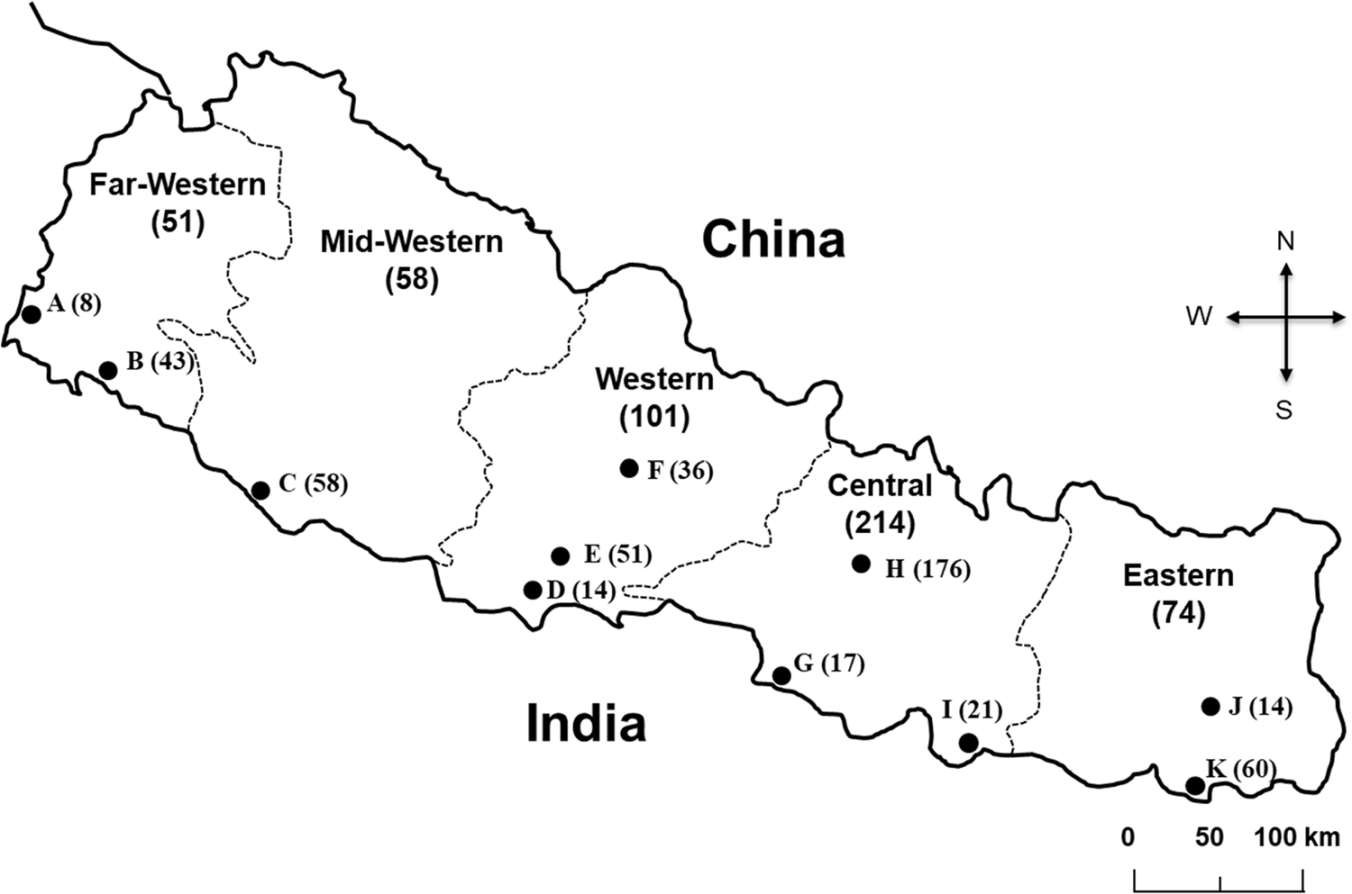
Locations of the 11 MDR-TB treatment centers in Nepal from where MDR-MTB samples were collected: (**A**) Mahendranagar, (**B**) Dhangadhi, (**C**) Nepalgunj, (**D**) Bhairahawa, (**E**) Butwal, (**F**) Pokhara, (**G**) Birgunj, (**H**) Kathmandu, (**I**) Dhanusa, (**J**) Dharan and (**K**) Biratnagar. Sample numbers collected in each center: (Total).

The geographical location and sample size of all 498 MDR-MTB isolates is shown in Fig. 1. Classification of all the isolates in this study according to patients’ geographic location, ethnic group, age and sex is described in Table 1. The majority of the isolates (214, 42.9%) were from the central region, belonged to the mongoloid ethnic group (204, 40.96%) and were male (355, 71.2%) (Table 1).

**Table 1:** Distribution of MDR-MTB isolates according to its genotypic lineages and patient’s geographical region, ethnic group, sex and age.

Variable	n (%)
**Genotypic lineages**	
1 (Indo-Oceanic)	32 (6.4)
2 (Beijing)	241 (48.4)
3 (CAS/Delhi)	153 (30.7)
4 (Euro-American)	72 (14.4)
**Region**	
Eastern	74 (14.8)
Central	214 (42.9)
Western	101 (20.2)
Mid-Western	58 (11.6)
Far-Western	51 (10.2)
**Ethnic group**	
Mongoloid	204 (40.96)
Non-mongoloid	294 (59)
**Age group (years)**	
0–23	129 (25.9)
24–30	127 (25.5)
31–42	120 (24)
>42	122 (24.4)
**Sex**	
Male	355 (71.2)
Female	143 (28.7)

Among the 498 MDR-MTB isolates, DST of 459 isolates were characterized by both phenotypic (DST) as well as by Genotype MTBDR*plus* line probe assay whereas 39 isolates were characterized by the Genotype MTBDR*plus* assay only. Of the 459 isolates characterized by phenotypic DST, 393 isolates were resistant to three or more first-line anti-TB drugs and 66 isolates were resistant to isoniazid (INH) and rifampicin(RIF) only. All the 39 isolates characterized by MTBDR*plus* were resistant to INH and RIF.

### Genotypes of MDR-MTB isolates

Of the 498 spoligotype results, a total 78 different spoligotypes were identified. Of these, 56 spoligotypes for 458 (92%) of the isolates were already defined in spoligo international types (SITs), while 32 spoligotypes from 40 (8%) of the isolates had a new spoligotype patterns and were classified as orphan types. The orphan types that did not match to any known lineages in the SITVIT database were further characterized by large sequence polymorphism (LSP) for lineage confirmation (Table 2). Among the 56 already defined spologotypes patterns, common spoligotypes were SIT 1, Beijing type (n = 239, 47.9%); SIT 26, CAS1_DELHI (n = 54; 10.8%); SIT 53, T1 (n = 33; 6.6%); SIT 599, CAS (n = 12; 2.4%) and SIT 11, EAI3_IND (n = 10; 2.0%), representing 69.7% of all isolates (Table 2). Finally, after lineage determination from spoligotyping and LSP, the most frequent lineage among MDR-MTB isolates was lineage 2 (Beijing family), with prevalence of 241 (48.4%) and followed by lineage 3 (CAS/Delhi family) with 153 (30.7%). Lineage 4 (Euro-American lineage) was the third most prevalent (n = 73, 14.5%) followed by Lineage 1 (Indo-Oceanic lineage) (n = 32, 6.4%) (Tables 1 and 2).

**Table 2:** Distribution of *Mycobacterium tuberculosis* lineages and spoligotype families among the MDR-TB in Nepal (n: 498).

Lineage*	SIT^†^	Clade	spoligo type pattern (spacer 1–43)	n	Genotyping
1	11	EAI3_IND	■■□□■■■■■■■■■■■■■■■■■■■■■■■■■□□□□■□■■□□□■■■■	10	spoligotype
1	138	EAI5	■■■■■■■■■■■■■■■■■■■■■■■■■■■■□□□□■□■■■■■□□□□	7	spoligotype
1	126	EAI5	■□□■■■■■■■■■■■■■■■■■■■■■■■■■□□□□■□■■■■■■■■■	5	spoligotype
1	1435	EAI1_SOM	■■■■■■■□□■■■■■■■■■■■■■■■■■■■□□□□■□■■■■■□■■■	1	spoligotype
1	48	EAI1_SOM	■■■■■■■■■■■■■■■■■■■■■■■■■■■■□□□□■□■■■■■□■■■	1	spoligotype
1	236	EAI5	□□□■■■■■■■■■■■■■■■■■■■■■■■■■□□□□■□■■■■■■■■■	1	spoligotype
1	763	EAI5	■■■■■■■■■■■■■□□□□□□■■■■■■■■■■□□□□■□■■■■■□□□□	1	spoligotype
1	2148	CAS	■■■□□□□□□□□□□□□□□□□□□□□□□□□□□□□□□□□□□□□□□□□	2	LSP^‡^
1	27	U(CAS_ANCESTOR?)	■■■□□□□■■■■■■■■■■■■■■■□□■■■■■■■■■□□□□■■■■■■	1	LSP
1	—	orphan	■■■■■■■■■■■■■■■■■■■■■■■■■■■■□□□□■□□□■■■□□□□	1	LSP
1	—	orphan	□□■□□□□■■■■■■■■■■■■■■■■■■■■■□□□□■□■■■■■□■■■	1	LSP
1	—	orphan	■■■■■■■■■■■■■■■■■■■■■■■■■■■■□□□■■□■■■■■■■■■	1	LSP
Subtotal				32	
2	1	Beijing	□□□□□□□□□□□□□□□□□□□□□□□□□□□□□□□□□□■■■■■■■■■	239	spoligotype
2	1168	Beijing	□□□□□□□□□□□□□□□□□□□□□□□□□□□□□□□□□□■■■■□□■■■	1	spoligotype
2	265	Beijing	□□□□□□□□□□□□□□□□□□□□□□□□□□□□□□□□□□■■□■■■■■■	1	spoligotype
Subtotal				241	
3	26	CAS1_DELHI	■■■□□□□■■■■■■■■■■■■■■■□□□□□□□□□□□□■■■■■■■■■	54	spoligotype
3	599	CAS	■■■□□□□■■■■■■■■■■■■□□□□□□□□□□□□□□□□□■■■■■■■	12	spoligotype
3	288	CAS2	■■■□□□□□□□■■■■■■■■■■■■□□□□□□□□□□□□■■■■■■■■■	6	spoligotype
3	357	CAS	■■■□□□□■■■■■■■■■■■■■■■□□□□□□□□□□□□□□■■■■■■■	6	spoligotype
3	2147	CAS1_DELHI	■■■□□□□■■■■■■■■■■■■■■■□□□□□□□□□□□□■■□□□□□■■	5	spoligotype
3	471	CAS1_DELHI	□■■□□□□■■■■■■■■■■■■■■■□□□□□□□□□□□□■■■■■■■■■	4	spoligotype
3	1312	CAS1_DELHI	■■■□□□□■■■■■■■■■■■■■■■□□□□□□□□□□□□■■□□■□■■■	4	spoligotype
3	22	CAS	■■■□□□□■■■■■■■■■■■■□□□□□□□□□□□□□□□■■■■■■■■■	4	spoligotype
3	427	CAS1_DELHI	■■■□□□□■■■■■□□□■■■■■■■□□□□□□□□□□□□■■■■■■■■■	3	spoligotype
3	25	CAS1_DELHI	■■■□□□□■■■■■■■■■■■■■■■□□□□□□□□□□□□■■□□■■■■■	3	spoligotype
3	142	CAS	■■■□□□□■■■■■■■■■■■■■■□□□□□□□□□□□□□■■■■■■■■■	3	spoligotype
3	486	CAS	■■■□□□□■■■■■■■■■■■■■■■□□□□□□□□□□□□□□□■■■■■■	3	spoligotype
3	428	CAS1_DELHI	■■■□□□□■■■■■■■■■■■■■■■□□□□□□□□□□□□■■□■■■■■■	2	spoligotype
3	429	CAS1_DELHI	■■■□□□□■■■■■■■■■■■■■■■□□□□□□□□□□□□■■■■■□■■■	2	spoligotype
3	1590	CAS	■■■□□□□■■■■■■■■■■■□■■■□□□□□□□□□□□□■■■■■■■■■	2	spoligotype
3	1093	CAS1_DELHI	■■■□□□□■■■■■■■■■■■■■□□□□□□□□□□□□□□■■■■■■■■■	2	spoligotype
3	1343	CAS1_DELHI	■■■□□□□■■■■■□■■■■■■■■■□□□□□□□□□□□□■■■■■■■■■	1	spoligotype
3	1314	CAS1_DELHI	■■■□□□□■■□■■■■■■■■■■■■□□□□□□□□□□□□■■□□■■■■■	1	spoligotype
3	1883	CAS1_DELHI	■■■□□□□■■■■■■■■■■■■■■■□□□□□□□□□□□□■■■■■■■□□	1	spoligotype
3	1947	CAS1_DELHI	■■■□□□□■■■■■■■■□□■■■■■□□□□□□□□□□□□■■■■■■■■■	1	spoligotype
3	2693	CAS1_DELHI	□■■□□□□■■■■■■■■■■■■■■■□□□□□□□□□□□□■■□□■■■■■	1	spoligotype
3	1968	CAS1_DELHI	■■■□□□□■■■■■■■■■■■■■■■□□□□□□□□□□□□■■■■■□□■■	1	spoligotype
3	1266	CAS	■■■□□□□□□■■■■■■■■■■■■■□□□□□□□□□□□□■■■■■■■■■	1	spoligotype
3	485	CAS	■■■□□□□■■■■■■■■■■■■□□□□□□□□□□□□□□□■■■■■■■■■	1	spoligotype
3	356	CAS	■■■□□□□■■■■■■■■■■■■■□□□□□□□□□□□□□□□■■■■■■■■	1	spoligotype
3	1151	CAS	■■■□□□□□□□■■■■■■■■■■■■□□□□□□□□□□□□□■■■■■■■■	1	spoligotype
3	203	CAS	■■■□□□□■■■■■■■■■■■■■■■□□□□□□□□□□□□□■■■■■■■■	1	LSP
3	1391	U	■■■■■■■■■■■■■■■■■■■■■□□□□□□□□□□□□□■■□■■■■■■	1	LSP
3	1089	CAS	■□□□□□□■■■■■■■■■■■■■■■□□□□□□□□□□□□■■■■■■■■■	1	LSP
3	—	orphan	■■■□□□□■■■■■■■■■■□□■■■□□□□□□□□□□□□■■■■■■■■■	2	LSP
3	—	orphan	■■■□□□□■■■■■■■■■■■■■■□□□□□□□□□□□□□□□□□□□□□□	2	LSP
3	—	orphan	■■■□□□□□□□■■■■■■■■■■■■□□□□□□□□□□□□□■■■■■□□□	2	LSP
3	—	orphan	■■■□□□□□□□■■■■■■■■■■■□□□□□□□□□□□□□□□□■■■■■■	2	LSP
3	—	orphan	■■■□□□□■■■■■■■■■■■□■■□□□□□□□□□□□□□□□■■■■■■■	1	LSP
3	—	orphan	■■■□□□□■■■■■□□■■■■■■■■□□□□□□□□□□□□■■■■■■■■■	1	LSP
3	—	orphan	■■■□□□□■■■■■■□■■■■■■□□□□□□□□□□□□□□■■■■■■■■■	1	LSP
3	—	orphan	■■□□□□□□□■■■■■■■■■■■■■□□□□□□□□□□□□■■■■■■■■■	1	LSP
3	—	orphan	■■■■■■■■■■■■■■■■■■■■■□□□□□□□□□□□□□□□□□□■■■■	1	LSP
3	—	orphan	■■■□□□□■■□■■■■■■■■■■■■□□□□□□□□□□□□□□■■■■■■■	1	LSP
3	—	orphan	□□■□□□□■■■■■■■■■■■■■■■□□□□□□□□□□□□■■■■■■■■■	1	LSP
3	—	orphan	■■■□□□□■■■■■■■■■□□□□□□□□□□□□□□□□□□□■■■■■■■■	1	LSP
3	—	orphan	■■■□□□□■■■■■□□■■■■■■■■□□□□□□□□□□□□■■■□■■■■■	1	LSP
3	—	orphan	■■■□□□□■■□□□□□□□■■■■■□□□□□□□□□□□□□■■■■■■■■■	1	LSP
3	—	orphan	■■■□□□□■■■■■■■□■■■■■□□□□□□□□□□□□□□■■■■■■■■■	1	LSP
3	—	orphan	■■■□□□□■■■■■■□□□□□□□□■□□□□□□□□□□□□■■■■■■■■■	1	LSP
3	—	orphan	■■■□□□□■■■■■■□□□□□□□□□□□□□□□□□□□□□□□□■■□■■■	1	LSP
3	—	orphan	■■■□□□□■■■■■□■■■□□□■■■□□□□□□□□□□□□□□□□■■■■■	1	LSP
3	—	orphan	■■■□□□□□■■□■■■□■■■■■■■■□□□□□□□□□□□■■■■■■■■■	1	LSP
3	—	orphan	■■■□□□□□■■■■■■■■■■■■□□□□□□□□□□□□□□■■■■■■■■■	1	LSP
3	—	orphan	■■■□□□□□□□■■■■■■■■■■■■□□□□□□□□□□□□□□□□□□□□□	1	LSP
Subtotal				153	
4	53	T1	■■■■■■■■■■■■■■■■■■■■■■■■■■■■■■■■□□□□■■■■■■■	33	spoligotype
4	52	T2	■■■■■■■■■■■■■■■■■■■■■■■■■■■■■■■■□□□□■■■□■■■	7	spoligotype
4	655	H3	■□□■■■■■■■■■■■■■■■■■■■■■■■■■■■□■□□□□■■■■■■■	4	spoligotype
4	42	LAM9	■■■■■■■■■■■■■■■■■■■■□□□□■■■■■■■■□□□□■■■■■■■	4	spoligotype
4	194	LAM2-LAM4	■■□■■■■■■■■■□■■■■■■■□□□□■■■■■■■■□□□□■■■□■■■	2	spoligotype
4	628	T1	■■■■■■■■■■■■■■■■■■■■■■■■■■■■■■■■□□□□■■■■■□□	1	spoligotype
4	1324	T1	■■■■■■■■■■■■■■□□□□□□□■□□■■■■■■■■□□□□■■■■■■■	1	spoligotype
4	1077	T2	■■■■■■■■■■■■■■■■■■□■■■■■■■■■■■■■□□□□■■■□■■■	1	spoligotype
4	1745	T3	■■■■■■□■■■■■□■■■■■■■■■■■■■■■■■■■□□□□■■■■■■■	1	spoligotype
4	119	X1	■■■■■■■■■■■■■■■■■□■■■■■■■■■■■■■■□□□□■■■■■■■	1	spoligotype
4	137	X2	■■■■■■■■■■■■■■■■■□■■■■■■■■■■■■■■□□□□■■□□□□■	1	spoligotype
4	92	X3	■■■□□□□□□□□□■■■■■□■■■■■■■■■■■■■■□□□□■■■■■■■	1	spoligotype
4	50	H3	■■■■■■■■■■■■■■■■■■■■■■■■■■■■■■□■□□□□■■■■■■■	1	spoligotype
4	20	LAM1	■■□■■■■■■■■■■■■■■■■■□□□□■■■■■■■■□□□□■■■■■■■	1	spoligotype
4	176	LAM6	■■■■■■■■■■■■□■■■■■■■□□□□■■■■□■■■□□□□■■■■■■■	1	spoligotype
4	—	Orphan	■■■■■■■■■■■■■■■■■□□□■■■■■■■■■■■■□□□□■■□□□□■	5	LSP
4	—	Orphan	■■■■■■■■■■■■■■■■■■■■■□□□■□□□□□□■□□□□■■■□■■■	1	LSP
4	—	Orphan	■■□■■■■■■■■■□■■■■□□□■■■■■■■■■□□■□□□□□□□□□□□	1	LSP
4	—	Orphan	■■■■■■■■■■■■■■■■□■■■■■■■■■■■■■■■□□□□■■□□□□■	1	LSP
4	—	Orphan	■■□□□□□□□■■■■■■■■□■■■■■■■■■■■■■■□□□□■■■■■■■	1	LSP
4	—	Orphan	■■■■■■■■■■■■■■■■■■□■■□■■■■■■■■■■□□□□■■■□■■■	1	LSP
4	—	Orphan	■■□□□□□□■■■■■■□□□□□□□■□□■■■■■■■■□□□□■■■■■■■	1	LSP
4	—	Orphan	■■■■■■■■■■■■■■■■■■■■■■■■■■■□□■■■□□□□■■■■■■■	1	LSP
Subtotal				72	

*Lineage 1: Indo-Oceanic, Lineage 2: East Asian (includes Beijing strains), Lineage 3: Delhi/CAS, Lineage 4: Euro-American lineage.

SIT^†^, Spoligotype International Type according to definition SITVIT web database (http://www.pasteur–guadelope.fr.8081/SITVIT_ONLINE).

LSP^‡^, Large sequence polymorphism.

### Association of different MDR-MTB lineages with geographic distribution and patient’s characteristics

**Figure 2:**
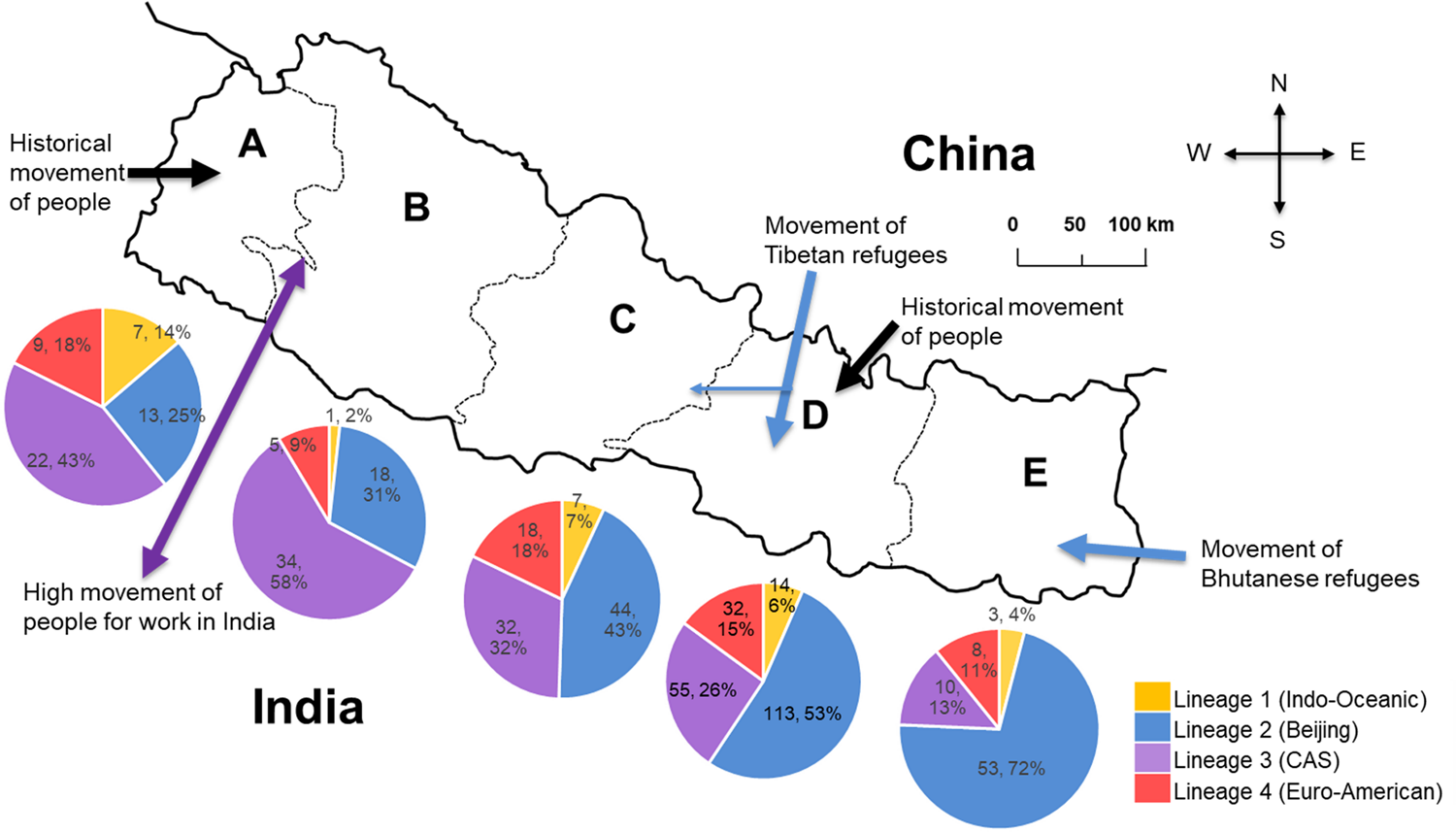
Geographical distribution of different lineages of MDR-MTB isolates in different geographic regions of Nepal: (**A**) Far-Western development region, (**B**) Mid-Western development region, (**C**) Western development region, (**D**) Central development region, (**E**) Eastern development region. Each line represents routes of people movement in Nepal as indicated. The distribution of lineages in each geographic region is represented in corresponding pie-chart as indicated.

Different lineages of MDR-MTB isolates were found to be significantly associated (P < 0.0001) with different geographical regions of the country (Table 3). The Lineage 2, Beijing family was the most prevalent in the eastern region with (n = 53, 72%) and its prevalence showed a decreasing westward pattern of distribution towards western Nepal (Fig. 2). The Lineage 3 was most prevalent in the mid-western (n = 34, 58%) and far-western regions (n = 22, 42%), with its prevalence showing a gradual decrease towards the eastern region of Nepal (Fig. 2). The other lineages were less prevalent and were evenly distributed throughout the country (Fig. 2).

**Table 3:** Association of genotypic lineages of MDR-MTB isolates with patient’s geographical region, ethnic group, sex and age.

MDR-TB patient’s characteristics	Lineage 1 (Indo-Oceanic)	Lineage 2 (Beijing)	Lineage 3 (CAS/Delhi)	Lineage 4 (Euro-American)	chi-square	*P*-value
	n (%)					
**Region**					55.05	<0.0001
Eastern Region	3 (4.1)	53 (71.6)	10 (13.5)	8 (10.8)		
Central Region	14 (6.6)	113 (52.8)	55 (25.7)	32 (15)		
Western Region	7 (6.9)	44 (43.6)	32 (31.7)	18 (17.8)		
Mid-Western Region	1 (1.7)	18 (31)	34 (58.6)	5 (8.6)		
Far Western Region	7 (13.7)	13 (25.5)	22 (43.1)	9 (17.6)		
**Ethnic group**					9.02	0.028
Mongoloid	10 (4.9)	115 (56.4)	53 (26)	26 (12.7)		
Non-Mongoloid	22 (7.5)	126 (42.9)	100 (34)	46 (15.6)		
**Age group (years)**					19.31	0.022
0–23	6 (4.7)	70 (54.3)	31 (24)	22 (17.1)		
23–30	5 (3.9)	71 (55.9)	39 (30.7)	12 (9.4)		
31–42	8 (6.7)	48 (40)	40 (33.3)	24 (20)		
>42	13 (10.7)	52 (42.6)	43 (35.2)	14 (11.5)		
**Sex**					16.34	0.0009
Male	27 (7.6)	163 (45.9)	123 (34.6)	42 (11.8)		
Female	5 (3.5)	78 (54.5)	30 (21)	30 (21)

As the Beijing family was the most prevalent lineage of MDR-MTB isolates in Nepal, we wanted to understand the association of this strain family with geographical region. The Beijing and non-Beijing family of isolates were significantly associated (P < 0.001) with different regions (Table 4). The odds ratio for presence of Beijing-family strains in the eastern region as compared to other regions was significantly lower than 1, indicating a high risk of TB infection with Beijing-family strains in eastern Nepal (Table 4).

**Table 4:** A univariate analysis of odds ratios for geographical distribution, ethnic group, age and sex between Beijing and Non-Beijing family of MDR-MTB isolates in Nepal.

Variance	Category	Beijing	Non-Beijing	Odds ratio (95% CI)	*P -* value
		n (%)			
Region	Eastern	53 (71.6)	21 (28.4)	1	—
	Central	113 (52.8)	101 (47.2)	0.443 (0.25–0.785)	0.005
	Western	44 (43.6)	57 (56.4)	0.306 (0.161–0.580)	<0.001
	Mid-Western	18 (31)	40 (69)	0.178 (0.084–0.378)	<0.001
	Far-Western	13 (25.5)	38 74.5)	0.125 (0.055–0.284)	<0.001
Ethnic group	Mongoloid	115 (56.4)	89 (43.6)	1	—
	Non-Mongoloid	126 (42.9)	168 (57.1)	0.580 (0.408–0.832)	0.003
Age group (years)	31–42	48 (40)	72 (60)	1	—
	>42	52 (42.6)	70 (57.4)	1.114 (0.668–1.859)	0.679
	0–23	70 (54.3)	59 (45.7)	1.780 (1.076–2.944)	0.024
	24–30	71 (55.9)	56 (44.1)	1.902 (1.147–3.155)	0.012
Sex	Male	163 (45.9)	192 (54.1)	1	—
	Female	78 (54.5)	65 (45.5)	1.414 (0.957–2.087)	0.081

*P* < 0.05 represents statistically significant difference.

Different lineages were also found to be significantly associated with patient’s age, ethnic group and sex (Table 3). Regarding age variance, the 25^th^percentile, median and 75^th^percentile of the variance were 23, 30, and 42 years old, respectively. Based on this calculation, the age variance was divided into four categories, 0–23, 24–30, 31–42, and >42.

For in-depth investigation, we divided strains into two groups, Beijing family and non-Beijing family, and found that the Beijing family was significantly associated with ethnic group and patient age (Table 3). Table 4 summarizes the univariate analysis of odds ratio between the Beijing family and non-Beijing family with different variances. In the final regression model, overall the predictors of geographical distribution and age was found to be associated with genetic diversity between Beijing and non-Beijing family MDR-TB cases (Wald = 32.174; 14.047, P < 0.05).

## Discussion

To our knowledge, this is the first investigation of genetic diversity of MDR-TB isolates covering all MDR-TB treatment centers and sub centers of the national TB program in Nepal. A diverse population of MTB was observed, showing 78 different spoligotype-based genotypes. About half of the isolates (241, 48.4%) belonged to Lineage 2, Beijing family and about one third (153, 30.7%) mapped to the Lineage 3, CAS/Delhi family. These 2 families of MTB accounted to (394, 79.1%) of the total population of MDR-MTB isolates indicating their epidemic spread in Nepal. Although, the previous study showed that CAS/Delhi family was the most prevalent MTB lineage in non MDR-TB Nepal^12^, our study demonstrates that the Beijing family is the most prevalent MDR-MTB lineage in Nepal.

The prevalence of Beijing family in MDR-TB in our study was higher than reported in India (35%)^14^ but lower than reported in China (75%)^15^, Vietnam (71%)^16^ and Thailand (72.4%)^17^. Nepal is surrounded by two major TB burden countries, China (including Tibet) to the north where Beijing family is dominant^7,18,19^ and India to the east, west and south where the CAS/Delhi family is dominant^6,20^. Despite the present situation of an open border, frequent people-to-people contact with India, and the dominance of CAS/Delhi family in non MDR-MTB strains^12^, we found the Beijing family to be the most prevalent in MDR-MTB strains in Nepal. The association of Beijing family is frequently associated with MDR-TB in Asia^10,11,15–17^ and our findings suggest that Beijing family is also the leading cause of MDR-TB in Nepalese population.

MTB lineages were significantly associated with geographic regions (Tables 3–5), with the Beijing family predominant in the eastern and central regions (Fig. 2). The eastern, central and the Himalayan regions of Nepal saw historical movements of the mongoloid ethnic group from the north and north-east (China including Tibet)^21^ (Fig. 2). Nepal also witnessed the movement of Tibetan refugees who mostly settled in the capital Kathmandu in the central region^22^ and while in the early 1990s Bhutanese refugees settled in the eastern Nepal^23^ (Fig. 2). People from Dharana city in eastern Nepal used to frequently travel to Hong Kong, China to work as members of the British Gurkha Army^24^. There is still frequent movement of people between the eastern region of Nepal and north-eastern parts of India where the Beijing family is dominant^25^. Since, MTB is suggested to have co-expanded in line with human migration^26^, these phenomena might explain the establishment of the Beijing family in eastern and central region and might be one of reasons for the higher chance of isolating the Beijing family in eastern Nepal (Tables 4–5). The Beijing family was the dominant genotype in the central region that includes Kathmandu city from where majority of isolates were collected (Fig. 1, Table 1). People of all ethnic groups from all over the country live and travel to Kathmandu. This observation may indicate that the Beijing family is the dominant or an emerging MDR-MTB lineage in Nepal.

**Table 5:** Final logistic regression model of genetic diversity of Beijing and non-Beijing family MDR-TB and predictor variables.

Variable	B	SE	Wald	Sig	Exp B (95% CI)
Constant	0.899	0.335	6.407	0.011	2.456
Region (RG)*			32.174	0.000	
RG (Central)	−0.954	0.306	9.721	0.002	0.385 (0.212–0.702)
RG (Western)	−1.408	0.343	16.902	0.000	0.245 (0.125–0.479)
RG (Mid-Western)	−1.891	0.405	21.803	0.000	0.151 (0.068–0.334)
RG (Far-Western)	−2.043	0.436	21.920	0.000	0.130 (0.055–0.305)
Ethnic (Non-Mongolian)	−0.323	0.211	2.345	0.126	0.724 (0.479–1.095)
Sex (Male)	0.42	0.215	0.38	0.845	1.043 (0.685–1.588)
Age (AGP)*			14.047	0.003	
AGP (>42)	−0.128	0.278	0.210	0.647	0.880 (0.510–1.519)
AGP (0–23)	0666	0.272	6.003	0.014	1.947 (1.143–3.319)
AGP (24–30)	0.653	0.273	5.713	0.017	1.921 (1.125–3.280)

*Parentheses; Reference: Region (RG)- Eastern region; Age (AGP)- 31–40.

We observed a high prevalence of CAS/Delhi family in mid- and far-western regions (Table 3, Fig. 2). This lineage may be a historically predominant strain in these regions due to the historic movement of people from northern India^21^ and the frequent movement of people from that area to India for work and business (Fig. 2).

We observed that lineages were significantly associated with patients’ ethnic group, sex and age (Table 3). In order to understand how the Beijing family might have interacted with these patient’s characteristics, we compared these patient parameters between Beijing and non-Beijing family strains and found that ethnic group and age were significantly associated (Table 3). Univariate analysis of odds analysis indicated that the isolation of Beijing family MTB strains from Mongoloid people was significantly higher than for other ethnic groups (Table 4). Mongoloid people have migrated to Nepal from Northeast Asia including Tibet^21^ (Fig. 2) where the Beijing family is the dominant^7,18,19^. As such, the association of MTB with human migration^26^ and recent evidence of Southern East Asian origin and historical co-expansion of the Beijing family with migration of people from East Asia, especially the Chinese population^27^ might provide evidence for the association of the Beijing family with mongoloid ethnic group. However, non-significant association of lineages of MTB with ethnic group from multivariate analysis (Table 5) suggest that Beijing family is not limited to mongoloid ethnic group and is successfully transmitting in Nepalese population.

The odds ratio of 1.902 and 1.919 for isolation of the Beijing lineage strains from persons aged 24–30 years is almost double that of those between 31 and 42 years (Tables 4–5). Similarly, a high odds ratio of 1.78 and 1.942 in the younger age group (0–23 years) suggests that younger age groups are with greater risk of infection from circulating and highly transmissible and circulating Beijing family MDR-TB due to their greater mobility and frequent public contacts^28^. The identification of a higher risk of MDR-TB based on age is an important finding from this study, and this may help to control MDR-TB in a resource-limited country like Nepal.

Despite a skewed male to female ratio of 2.48:1 (355:143) in this study population, a significant association was not seen between sex and Beijing family (Table 4); however, the proportion of lineage 2 isolates was higher in females (54.5%, 78/143) than in males (45.9%, 163/355). This finding is also consistent with previous findings from Nepal^12^.

Apart from the association of the above variables with MDR-MTB lineages, other variables could have played a role in lineage dominance; for example, we could not study the patients’ socio-economic status, their locations and frequency of social gatherings, family tuberculosis history, and other medical history such as HIV status and travel history. We have observed that many of the TB patients have relatively low socio-economic condition, consume alcohol and frequently use public transport to visit MDR-TB treatment centers. These factors could also play role in lineage distribution and potentially favor expansion of the Beijing family of MDR-TB in Nepal.

In this work, we studied the relationship of spoligotyping-based genotypes of MDR-MTB isolates with patients’ characteristics. Because of limited strain discriminating power of provided by spoligotying, we could not define the transmission dynamics of MDR-MTB isolates. In the future, we would like to perform, and recommend the combination of spoligotyping with mycobacterial interspersed repetitive unit–variable number tandem repeat typing (MIRU-VNTR) or the use of whole genome sequencing for comprehensive understanding disease dynamics of MDR-TB in Nepal.

Our results suggest that, despite the presence of diverse genotypes of MDR-MTB strains in Nepal, the Beijing family is the dominant MTB genotype linked to MDR-TB in Nepal. Our study suggests that Beijing-family strains are expanding by virtue of their distribution in high population densities, ethnic groupings, and, the higher mobility of younger age groups. We believe that the Beijing family is driving MDR-TB in Nepal and assume that its role in association with MDR-TB will increase in the future. Thus, we consider that the Beijing family of MDR-MTB is an emerging threat for the control of MDR-TB in Nepal. Recently, Nepal has strengthened its capacity to diagnose and manage MDR-TB by performing DST for all MDR-TB samples, expanded use of GeneXpert and introduced molecular diagnosis facility in Kathmandu. Additionally, we suggest introducing genotyping as a component of regular MDR-TB diagnosis in Nepal. The correct treatment of MDR-TB is of course the main clinical issue, regardless of the lineage of the causative MDR-TB strains. However, within the present context of the limited resources, we would like to suggest initiating intensive care and management of at least Beijing MDR-TB patients and some critical cases from other lineages, if intensive management of all MDR-TB patients is difficult. Some of the intensive management options would be isolated treatment and epidemiological surveillance of these patients, which may help to find new cases from their epidemiological links, understand risk factors and develop strategies for control and management of MDR-TB in Nepal. With the advancement of molecular laboratory science, rapid diagnostic biochemical and molecular methods such as whole genome sequencing will be used for TB diagnosis and management. Nepal should therefore prepare in advance to take advantage of upcoming innovations for the control and management of MDR-TB in Nepal.

## Materials and Methods

### Study population and settings

A total of 877 category II failures or DST confirmed MDR-TB patients (from new patients, category I failures, relapse treatment after lost to follow up and MDR contacts) were registered in the national MDR-TB program from April 2009 to Mar 2013. Among those registered patients, the majority were cat II failures without DST analysis because during the study period culture and DST facilities were not available outside the Kathmandu valley. After registration of patients, an initial sample was sent to Kathmandu for culture and DST. All positive cultures were then tested for phenotypic DST and Genotype MTBDR*plus* (Hain Life Science) for identification of MTB complex and resistance to INH and RIF. Finally, from the 877 registered patients, 498 patients’ samples that were confirmed to be MDR-MTB from culture, DST of INH and RIF and genotyping results were selected for analysis in this study (Fig. 1). Because of the issue of long sample transportation time and ongoing treatment, some samples were culture contaminated and negative. Additionally, a few samples were identified to be nontuberculosis mycobacteria and were excluded from our study. Thus, our sample collection strategy was purposive sampling to collect MDR-MTB isolates.

Sample processing, culture, DST and DNA extraction were performed at the German Nepal TB Project (GENETUP), National Reference Laboratory, Kathmandu. Samples were processed at bio-safety level 3 facility of GENETUP under direct supervision of NTP and WHO. Spoligotyping and Large Sequence Polymorphism (LSP) testing were performed at Hokkaido University, Japan.

### Drug susceptibility test (DST)

DST for isoniazid (INH, Cat No. 2261/0801; FatolArzneimittel GmbH, Schiffweiler, Germany), rifampicin (RIF, Cat No. 004030; Fatol), streptomycin (STR, Cat No. S6501; Sigma–Aldrich, St. Louis, MO), and ethambutol (EMB, Cat No. 1237/0806; Fatol) were performed by the indirect proportional method on Lowenstein-Jensen medium with critical concentrations of 0.2 μg/ml INH, 40 μg/ml RIF, 4 μg/ml STR and 2 μg/ml EMB^29,30^.

### DNA extraction

DNA was prepared for PCR following the manufacturers protocol using the Genotype MTBDR*plus*Ver 2 (HainLifescienceNehren, Germany)^31^. Briefly, colonies from positive cultures were suspended in 300 µl of DNA free distilled water and heated for 20 min at 95 °C. The heated sample was incubated in an ultra-sonic water bath for 15 min and centrifuged for 5 min at 10,000 × g. DNA from the supernatant was used for PCR amplification.

### GenotypeMTBDR*plus* Assay

Genotype MTBDR*plus* assay was performed according to the manufactures recommend protocol using the Genotype MTBDR*plus*Ver 2. Briefly,target DNA was amplified using a multiplex PCR mastermix(50 µl /tube) and theHot star*Taq*DNA Polymerase (Qiagen, Crawley, UK). Results were evaluated and interpreted following the manufacturers instruction^32^.

### Spoligotyping

Spoligotyping of MTB clinical isolates was performed as described previously^4^. Briefly, the DR region was amplified with a pair of primers, and the resulting PCR products were hybridized to a set of 43 spacer-specific oligonucleotide probes, which were immobilized in the membrane and hybridized DNA fragments were detected. Results were compared with the SITVITWEB database^33^ (http://www.pasteur-guadeloupe.fr:8081/SITVIT_ONLINE/) to determine spoligointernational Type (SIT). Spoligotyping patterns that were not identified by SITVITWEB were further analyzed by LSP^34^.

### Large sequence polymorphism (LSP)

A PCR-based technique was applied for the identification of isolates belonging to orphan or new spoligotypes using the specific primers for the expected regions of difference (RD) for each lineage as described^34^.

### Statistical analysis

Microsoft excel was used to analyze the data. Pearson’s chi square test was used to compare different variances. A *p* value less than 0.05 was considered statistical significant. Univariate analysis was performed to calculate the odds ratio with 95% confidence interval to understand risk of MDR-TB between Beijing and non-Beijing family in different situations. Furthermore, for multivariant analysis patient’s characteristics and outcome of MDR-MTB lineages were entered in the SPSS software version 19 (http://www01.ibm.com/software/analytics/spss/). Logistic regression was used for multivariate analysis of potential predictors of Beijing and Non-Beijing family MDR-TB; with P < 0.05 used as a threshold for statistically significant interactions. The default “enter” method was used.

### Ethics statement

This study proposal was submitted to Nepal Health Research Council (NHRC) with register no 136/2013 on 20^th^ Sep 2013 and approved by Ethical Review Board on 29^th^ Nov 2013.

## Data Availability

Data generated or analyzed during this study are included in this published article.

## References

[1] World Health Organization. Global tuberculosis report 2017. (Geneva, Switzerland: WHO, 2017), http://www.who.int/tb/publications/global_report/en/ (Accessed on 2017/12/18).

[2] National Tuberculosis Program, Nepal. Annual report 2015. http://www.nepalntp.gov.np/index.php?view=publication (Accessed on 2017/12 4) (2015).

[3] Shrestha L, Jha KK, Malla P (2010). Changing tuberculosis trends in Nepal in the period 2001-20018. Nepal. Med. Coll. J..

[4] Kamerbeek J (1997). Simultaneous detection and strain differentiation of Mycobacterium tuberculosis for diagnosis and epidemiology. J. Clin. Microbiol..

[5] Brudey K (2006). *Mycobacterium tuberculosis* complex genetic diversity: mining the fourth international spoligotyping database (SpolDB4) for classification, population genetics and epidemiology. BMC. Microbiol..

[6] Singh J (2015). Genetic diversity and drug susceptibility profile of Mycobacterium tuberculosis isolated from different regions of India. J.Infect..

[7] Pang Y (2012). Spoligotyping and drug resistance analysis of Mycobacterium tuberculosis strains from national survey in China. PloS One..

[8] Liu Y (2017). The study on the association between Beijing genotype family and drug susceptibility phenotypes of Mycobacterium tuberculosis in Beijing. Sci. Rep..

[9] Yuan L (2015). There is no correlation between sublineages and drug resistance of *Mycobacterium tuberculosis* Beijing/W lineage clinical isolates in Xinjiang, China. ‎Epidemiol. Infect..

[10] Merker M (2005). Evolutionary history and global spread of the *Mycobacterium tuberculosis* Beijing lineage. Nature. Genet..

[11] Barnes PF, Cave MD (2003). Molecular epidemiology of tuberculosis. N. Engl. J Med..

[12] Malla B (2012). First Insights into the phylogenetic diversity of *Mycobacterium tuberculosis* in Nepal. PLoS One..

[13] Shah Y (2017). High diversity of multidrug-resistant *Mycobacterium tuberculosis* Central Asian Strain isolates in Nepal. Int. J. Infect. Dis..

[14] Almeida D (2005). High incidence of the Beijing genotype among the multidrug-resistant isolates of *Mycobacterium tuberculosis* in a tertiary care center in Mumbai, India. Clin. Infect. Dis..

[15] Zhang D (2014). Genetic diversity of multidrug-resistant tuberculosis in a resource-limited region of China. Int. J. Infect. Dis..

[16] Buu TM (2009). The Beijing genotype is associated with young age and multidrug-resistant tuberculosis in rural Vietnam. Int. J. Tuberc. Lung. Dis..

[17] Disratthaki A (2015). Genotypic diversity of multidrug-, quinolone- and expensively drug-resistant *Mycobacterium tuberculosis* isolates in Thailand. Infect. Genet. Evol..

[18] Chan MY (2001). Seventy percent of the *Mycobacterium tuberculosis* isolates in Hong Kong represent the Beijing genotype. Epidemiol. Infect..

[19] Dong H (2012). Genetic Diversity of *Mycobacterium tuberculosis* isolates from Tibetans in Tibet, China. PLoS One..

[20] Singh UB (2007). Genetic biodiversity of *Mycobacterium tuberculosis* isolates from patients with pulmonary tuberculosis in India. Infect. Genet. Evol..

[21] Kansakar, V.B.S. History of Population Migration in Nepal. The Himalayan Review. 6(No. 5 & 6) (1973–74). http://104.236.33.185:8001/jspui/bitstream/123456789/25/1/History%20of%20Population%20Migration%20in%20nepal.pdf (Accessed on 2014/07/14).

[22] Tibet Justice Center. Tibet’s Stateless Nationals: Tibetan Refugees in Nepal. (2002). http://www.tibetjustice.org/reports/nepal.pdf (Accessed on 2014/07/14).

[23] International Organization for Migration. The Bhutanese refugees in Nepal. IOM Damak Nepal. http://www.peianc.com/sitefiles/File/resources/cultural_profiles/Bhutanese-Refugees-in-Nepal.pdf. (Accessed on 2014/07/14) (2008).

[24] Ale, S. Assessing the situation of Nepalese community in Hong Kong. Master’s thesis. Department of Asian and international studies, city university of Honk Kong. http://www.statistics.gov.hk/pub/B11200622012XXXXB0100.pdf (Accessed on 2018/01/10) (2013).

[25] Devi KR (2015). Genetic Diversity of Mycobacterium tuberculosis isolates from Assam, India: Dominance of Beijing family and discovery of two new clades related to CAS1_Delhi and EAI family based on spoligotyping and MIRU-VNTR typing. PLoS One..

[26] Comas I (2013). Out-of -Africa migration and neolithic co-expansion of *Mycobacterium tuberculosis* with modern humans. Nat. Genet..

[27] Luo T (2015). Southern East Asian origin and coexpansion of *Mycobacterium tuberculosis* Beijing family with Han Chinese. Proc. Nati. Acad. Sci. USA.

[28] Parwati I (2010). Possible underlying mechanisms for successful emergence of the *Mycobcterium tuberculosis* genotype strains. Lancet. Infect. Dis..

[29] World Health Organization. eneva. Guidelines for surveillance of drug resistance in tuberculosis. (Geneva, Switzerland, 2009). http://whqlibdoc.who.int/publications/2009/9789241598675_eng.pdf. (Accessed on 2014/07/17).

[30] Poudel A (2012). Molecular characterization of multidrug-resistant *Mycobacterium tuberculosis* isolated in Nepal. Antimicrob. Agents. Chemother..

[31] HainLifescience. GmbH GenoTypeMTBDR*plus* 1.0 product inserts. HainLifescience GmbH, Nehren, Germany. http://www.hain-lifescience.de/en/ (Accessed on 2014/07/17).

[32] Nikolayevskyy V (2009). Performance of the Genotype^®^ MTBDR*Plus* assay in the diagnosis of tuberculosis and drug resistance in Samara, Russian Federation. BMC. Clinical. Pathol..

[33] Demay C (2012). SITVITWEB – A publicly available international multimarker database for studying Mycobacterium tuberculosis genetic diversity and molecular epidemiology. Infect. Genet. Evol..

[34] Gagneux S (2006). Variable host–pathogen compatibility in *Mycobacterium tuberculosis*. Proc. Nati. Acad. Sci. USA.

